# First-principles study of sodium adsorption on defective graphene under propylene carbonate electrolyte conditions†

**DOI:** 10.1039/d2ra08168g

**Published:** 2023-02-14

**Authors:** Chol Ryu, Song-Bom Rim, Yong Kang, Chol-Jun Yu

**Affiliations:** a Chair of Computational Materials Design (CMD), Faculty of Materials Science, Kim Il Sung University PO Box 76 Pyongyang Democratic People's Republic of Korea cj.yu@ryongnamsan.edu.kp

## Abstract

Hard carbon (HC) has been predominantly used as a typical anode material of sodium-ion batteries (SIBs) but its sodiation mechanism has been debated. In this work, we investigate the adsorption of Na atoms on defective graphene under propylene carbonate (PC) and water solvent as well as vacuum conditions to clarify the sodiation mechanism of HC. Within the joint density functional theory framework, we use the nonlinear polarizable continuum model for PC and the charge-asymmetric nonlocally-determined local electric solvation model for water. Our calculations reveal that the centre of each point defect such as mono-vacancy (MV), di-vacancy (DV) and Stone–Wales is a preferable adsorption site and the electrolyte enhances the Na adsorption through implicit interaction. Furthermore, we calculate the formation energies of multiple Na atom arrangements on the defective graphene and estimate the electrode potential *versus* Na/Na^+^, verifying that the multiple Na adsorption on the MV and DV defective graphene under the PC electrolyte conditions is related to the slope region of the discharge curve in HC. This reveals new prospects for optimizing anodes and electrolytes for high performance SIBs.

## Abstract 2

First-principles simulations of Na cluster adsorption on defective graphene sheets under propylene carbonate conditions have been performed within joint density functional theory for revealing the mechanism of hard carbon for sodium-ion batteries.

## Graphical abstract

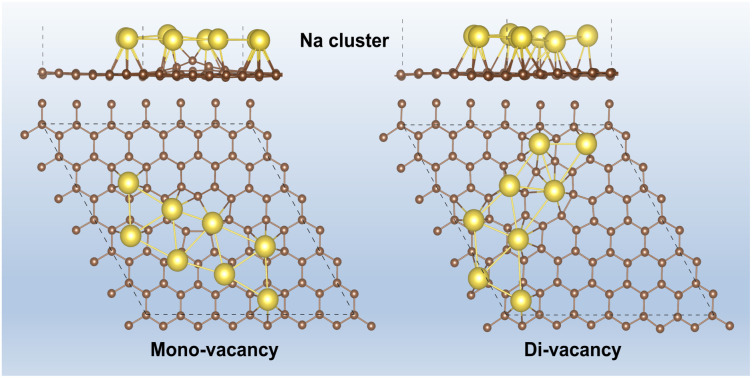

## 1 Introduction

Sodium-ion batteries (SIBs) are significantly attractive as potential large-scale energy storage devices for electric vehicles and intermittent renewable energy sources due to the high abundance and low cost of sodium resources.^1–3^ Remarkable successes have already been made in progressing the SIB technology towards high energy density and long-term cycling life.^4,5^ In this progress, the most important issue is to develop suitable electrode materials for SIBs, especially anode materials.^6–9^ In fact, graphite is a prototypical intercalation-type anode material for Li-ion batteries^10^ but does not accommodate Na properly in the first stage due to the larger ionic radius of the Na^+^ ion and weaker binding strength of Na–C than the Li^+^ ion.^11–15^ Although co-intercalation of Na^+^ ions with organic molecules was developed for use of graphite in SIBs,^16–19^ the specific capacity is still low, being below 100 mA h g^−1^.^20,21^

To resolve the problem, hard carbons (HCs) have been prepared as a promising anode material for SIBs by high-temperature pyrolysis of hydrocarbon precursors such as sugar, biomass and polymers under Ar or N_2_ flow.^22–28^ By constant efforts of many researchers, the anode performance of HCs has been continuously improved so as to reach the specific capacity over 400 mA h g^−1^, low electrode potential around 0.1 V and long-term cycling stability.^29,30^ Along with the enhancement of performance, understanding of structure and sodiation mechanism of HC has been provided.^31–35^ HC is composed of disordered graphitic domains with multi-layered graphene sheets and interstitial nano-pores, according to the well-accepted “house of cards” model suggested by Stevens and Dahn.^31^ Based on this structural model, the adsorption/intercalation/pore-filling has been proposed as credible sodiation mechanism. That is, Na ions were adsorbed on the surface of HC particles, intercalated into graphitic domains, and filled in nano-pores by adsorption on the pore wall and clustering inside the pore. In connection with the charge/discharge curve, it was accepted that the Na adsorption on the defect or edge sites of the pore wall is related with the slope region (above 0.1 V). The Na clustering on the defective pore wall resulted in the plateau capacity (below 0.1 V). The intercalation into the graphene sheets with the expanded interlayer distance over 3.75 Å contributed to both the slope and plateau capacities.^31,32,35^ However, the structural model and Na storage mechanism have been debated.^36,37^

First-principles materials modeling and simulations within the density functional theory (DFT) framework play a complementary role in identifying the detailed process of Na storage.^38–42^ Tsai *et al.*^41^ performed *ab initio* calculations of Na adsorption onto and intercalation into defective graphene sheets. They revealed that the vacancy defects such as mono-vacancy (MV) and di-vacancy (DV) enhance the Na intercalation into expanded and disordered graphene sheets due to the strong binding energy of Na–C by defects. The Na cluster adsorptions on pristine and DV-containing graphene have been investigated and reported as possible mechanism of pore-filling.^39,40^ Moreover, Youn *et al.*^38^ conducted the comprehensive DFT study, demonstrating that the nano-size Na clusters with 3–6 layers in the pore as well as island-shape small clusters on the pore wall are energetically stable to be formed for origin of the plateau capacity. Gueorguiev and co-workers clarified the aspects of interaction of atoms, molecules or nano-clusters with graphene sheets and their derivatives using DFT calculations.^43,44^ However, all the DFT calculations were performed under vacuum conditions and did not consider the influence of electrolyte on the performance. In this work, we investigate adsorption of Na ions and clusters on the graphene sheet with a defect of MV or DV or Stone–Wales (SW) in considering the effect of electrolyte, typically propylene carbonate (PC), by using joint density functional theory (JDFT) calculations.

## 2 Computational method

The first-principles calculations were performed in the JDFT framework as implemented in the JDFTx package.^45^ The electrolyte solvation effect was considered by using the nonlinear polarizable continuum model (PCM)^46^ for PC and the charge-asymmetric nonlocally-determined local electric (CANDLE) solvation model^47,48^ for water. As the material parameters of PC and water provided in the package, the bulk dielectric constants are 64 and 78.4, the optical-frequency dielectric constants are 2.02 and 1.77, and the dipole moment of each molecule are 2.95 and 0.92*e* bohr. The Perdew–Burke–Ernzerhof (PBE) functional within generalized gradient approximation (GGA)^49^ was adopted for the exchange–correlation energy. To take account of the van der Waals (vdW) interaction, the dispersion correction was added by using the DFT-D2 method.^50^ The ultrasoft pseudopotentials for elements were provided from GBRV library.^51^ Kinetic energy cutoffs for the plane wave basis set were 20 and 100 Ha for wave function and electron density, respectively. For the 6 × 6 × 1 supercells of graphene, the *k*-point meshes were set to 3 × 3 × 1 for structural optimizations and 4 × 4 × 1 for electronic structure calculations, respectively. In addition, the vacuum layer thickness along the normal direction was 15 Å, and filled by implicit solvation model, which contained 0.1 M NaClO_4_ sodium salt as solute and PC or water as solvent. These computational parameters ensure the energy convergence of 5 meV per atom (see Fig. S14† for convergence test). During the structural optimizations, the atoms were relaxed until the force on each atom converged to 0.1 mHa bohr^−1^.

## 3 Results and discussion

### 3.1 Adsorption of Na atom on defective graphene sheet

As a preliminary step, the lattice constants of graphite unit cell were determined by performing structural optimizations under the vacuum conditions. As discussed in our previous work,^52^ the lattice constants of *a* = 2.461 Å and *c* = 3.363 Å in good agreement with the experimental values (2.46 and 3.36 Å) could be obtained by adjusting the vdW parameters (*C*_6_ = 1.75 J nm^−6^, *R*_0_ = 1.642 Å) with DFT+D2 method implemented in the JDFTx code. With these vdW parameters, the interlayer binding energy was calculated to be −25.0 meV per atom, which is also in accord with the experimental value (−31 to − 52 meV). Using the determined lattice constants, we then constructed the 6 × 6 × 1 supercell for modeling graphene sheet, which was proven to be the most suitable size to simulate the point defect.^41^ On this supercell, a typical point defect such as MV, DV and SW was created and optimized by performing atomic relaxation (see Fig. S1†). The formation energy of defect was calculated by *E*_f_ = *E*_def-G_ − *E*_pris-G_ + *n*_C_*μ*_C_, where *E*_def-G_ and *E*_pris-G_ are the total energies of defective and pristine graphene sheets, respectively. *n*_C_ is the number of C atom removed from the pristine graphene, and *μ*_C_ is the chemical potential of carbon estimated by the total energy of graphite unit cell per atom. For the MV, DV, and SW defects, the calculated formation energies are 8.14, 7.61, and 5.33 eV, which agree well with the previous calculation and experimental values of 7.3–7.5, 7.2–7.9, and 4.5–5.3 eV, respectively.^41^ The calculations indicate that those vacancies are highly energetic defects and their formations are endothermic. Despite this, such vacancies were observed experimentally using transmission electron and scanning tunnelling microscopy.^53^

The Na adsorption on the surface of hard carbon and the pore wall is regarded as one of Na storage mechanisms as it is associated with the slope region of the charge/discharge curve. The HC surface and the pore walls are composed of graphitic domains, and therefore graphene model can be used to simulate them in this work. Moreover, it was reported that the number of graphene layers hardly affects the Na adsorption.^38^ Thus, instead of the graphite (0001) surface, it is admissible to use the graphene sheet for the studies of Na adsorption. Since the HC anode is in contact with electrolyte in SIBs, we considered the influence of electrolyte, typically PC and water, on the Na adsorption on graphene. The adsorption energy was estimated by using the following equation,

1 *E*_ad_ = *E*_Na-G_ − *E*_G_ − *μ*_Na_

where *E*_Na-G_ and *E*_G_ are the total energies of one Na adatom-adsorbed graphene and the free graphene, and *μ*_Na_ is the chemical potential of Na being equal to the total energy per atom of bcc Na bulk. For the pristine (defect-free) graphene, the adsorption energies of the Na adatom on the hollow site were calculated to be 0.05, −1.23, and −1.82 eV for vacuum, PC, and water environments respectively, as listed in Table 1. The distances of Na adatom above the graphene sheet *d*_Na-G_ were found to be 2.323, 2.486, and 2.744 Å under the vacuum, PC, and water conditions, respectively. This indicates that under vacuum conditions Na adsorption is unstable on the pristine graphene and only stable on the defective graphene in accordance with the previous works,^33,39,41^ whereas under electrolyte conditions Na is stable to be adsorbed on the perfect graphene sheet.

**Table 1:** Adsorption energy (*E*_ad_) and distance of Na adatom above the graphene sheet (*d*_Na-G_), and Lowdin charge states of Na (*Z*_Na_) and C atoms (*Z*_C_) upon Na adsorption at the N1 site, calculated with JDFTx code under vacuum, PC, and water conditions

Defect	Conditions	*E*_ad_ (eV)	*d*_Na-G_ (Å)	*Z*_Na_ (*e*)	*Z*_C_ (*e*)
Perfect	Vacuum	0.05	2.323	0.782	−0.030
	PC	−1.23	2.486	0.966	−0.005
	Water	−1.82	2.744	0.982	
MV	Vacuum	−1.46	2.221	0.843	−0.126
	PC	−2.34	2.360	0.914	−0.110
	Water	−2.68	2.486	0.944	−0.066
DV	Vacuum	−1.05	1.952	0.891	−0.101
	PC	−1.92	2.101	0.936	−0.090
	Water	−2.35	2.407	0.956	−0.066
SW	Vacuum	−0.21	2.164	0.880	−0.036
	PC	−1.38	2.284	0.960	−0.029
	Water	−1.89	2.590	0.975	−0.027

**Fig. 1:**
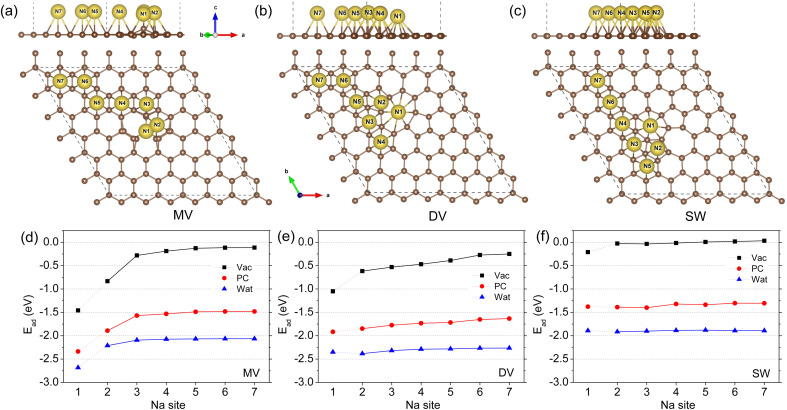
Optimized structures of one Na adatom adsorbed defective graphene sheets with a point defect of (a) mono-vacancy (MV), (b) di-vacancy (DV) and (c) Stone–Wales (SW) in side and top views under the vacuum conditions, and (d–f) the corresponding adsorption energy along the different adsorption sites from N1 to N7 calculated under the vacuum, PC and water conditions.

For the defective graphene with MV, DV, and SW point defects, the different hollow sites for one Na adatom adsorption, denoted as N_*i*_ (*i* = 1–7), are identified around the defect zone in accordance with the previous work,^41^ as shown in Fig. 1(a)–(c). For the three kinds of defective graphene sheets, the N1 site is just the centre of defect zone and the other hollow sites with increasing number are assigned to the sites as gradually moving away from the defect zone centre. Using eqn (1), the adsorption energies were calculated and plotted as a function of sites with increasing number, as shown in Fig. 1(d)–(f). Under the vacuum conditions, our calculations are almost identical to the previous work of Yamada and co-workers.^41^ On the MV, DV, and SW defective graphene sheets, the defect zone centre N1 sites were found to be the most stable si†tes with the lowest adsorption energies of −1.46, −1.05, and −0.21 eV and the smallest distances *d*_Na-G_ of 2.221, 1.952, and 2.164 Å, respectively (Table 1). As the Na adatom goes further away from the N1 site, the adsorption energy gradually approaches zero, indicating a decrease of the stability of Na adsorption. For the SW case, the values of *E*_ad_ on the other sites became positive, implying that the Na adsorption is not spontaneous (or exothermic) anymore but endothermic.

When including the electrolyte effect using the NaClO_4_ solute and PC or water solvent, the sodiation of graphene was found to occur more readily due to more negative values of Na adsorption energy. For the case of MV defective graphene, one can see similar tendencies along the adsorption sites to the case of vacuum conditions as shown in Fig. 1(d). For the DV and SW cases, the adsorption energies on the different sites were little changed as can be seen in Fig. 1(e) and (f). Furthermore, the favourable effect of water electrolyte on Na adsorption was found to be stronger than PC electrolyte due to the larger dielectric constant of water (78.4) than that of PC (64). Anyhow, the N1 hollow sites were the most stable adsorption sites for the three kinds of defective graphene in both vacuum and electrolyte environments.

**Fig. 2:**
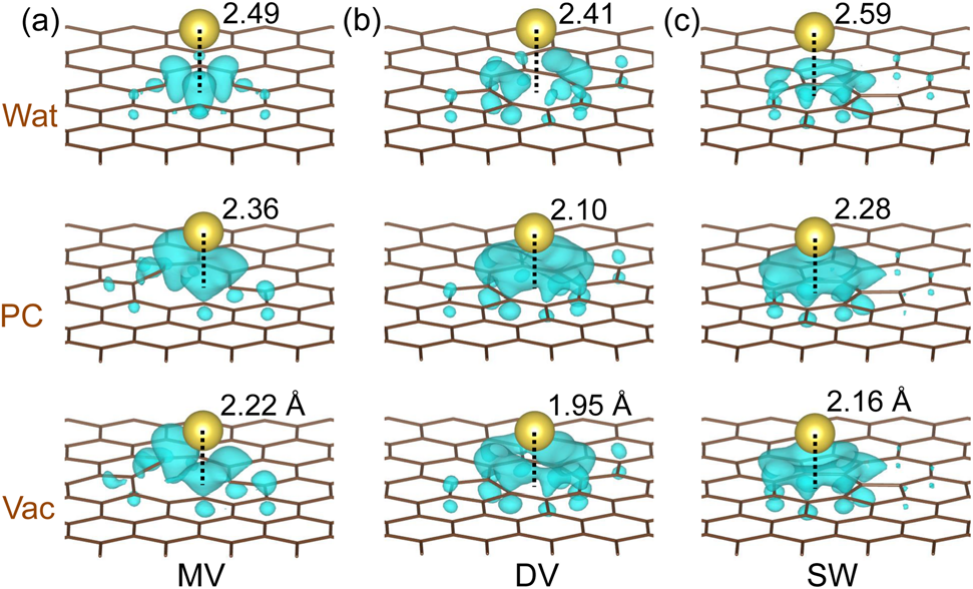
Isosurface view of the electronic density differences at the value of 0.0015|*e*| Å^−3^ upon Na adsorption at the N1 hollow site over the defective graphene with (a) MV, (b) DV, and (c) SW defect in the vacuum, PC and water solvent environment. Only the electron accumulation is shown. The distances of Na ion over the graphene plane are presented.

To get an insight into the charge transfer upon Na adsorption, the electronic density difference was obtained by subtracting the electron densities of the graphene sheet (*ρ*_G_) and sodium (*ρ*_Na_) from that of the Na-adsorbed graphene (*ρ*_Na-G_) as Δ*ρ* = *ρ*_Na-G_ − (*ρ*_G_ + *ρ*_Na_). Fig. 2 shows the calculated electronic density accumulation upon Na adsorption at the N1 hollow site (see Fig. S3† for the electronic density depletion). One can see that the valence electronic density has transferred almost totally from the Na atom to the bonding carbon atoms, indicating the ionic bonding character between the Na^+^ ions and carbons. The quantitative analysis of the charge transfer was provided by the Lowdin charge states of the atoms as listed in Table 1. Under the vacuum conditions, the Lowdin charges of Na ions are 0.843, 0.891, and 0.880 for MV, DV, and SW defects, which are larger than that for the perfect graphene 0.782. For the MV case, the positive charge of Na ion was increased as going vacuum to PC (0.914) and to water (0.944) solvent, confirming the enhancement of Na adsorption under the electrolyte conditions. For the DV and SW cases, similar tendencies were observed. Meanwhile, the negative Lowdin charges of carbons were decreased in magnitude as going from vacuum to PC and to water, indicating the weakening in Na–C ionic interaction in accordance with the enlargement of Na adatom distance over the graphene sheet. Therefore, it can be said that the enhancement of Na adsorption under the electrolyte environment is due to the implicit interaction between the Na atom and the electrolyte.

### 3.2 Adsorption of multiple Na atoms on defective graphene

For a high specific capacity of Na, it is required that HC must have a sufficiently high concentration of defect or adsorb multiple Na atoms on the defect.^38^ Therefore, we studied the adsorption of multiple Na atoms on the defective graphene sheet with a consideration of electrolyte effect. The number of Na atoms in the cluster was systematically increased from 2 to 8 on the 6 × 6 × 1 supercell of defective graphene. In order to evaluate the stability of adsorbed multiple Na atoms, we estimate the formation energy per Na atom as follows,2
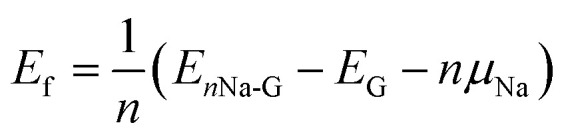
where *E*_*n*Na-G_ is the total energy of multiple Na atoms adsorbed graphene and *n* is the number of adsorbed Na atoms.

**Fig. 3:**
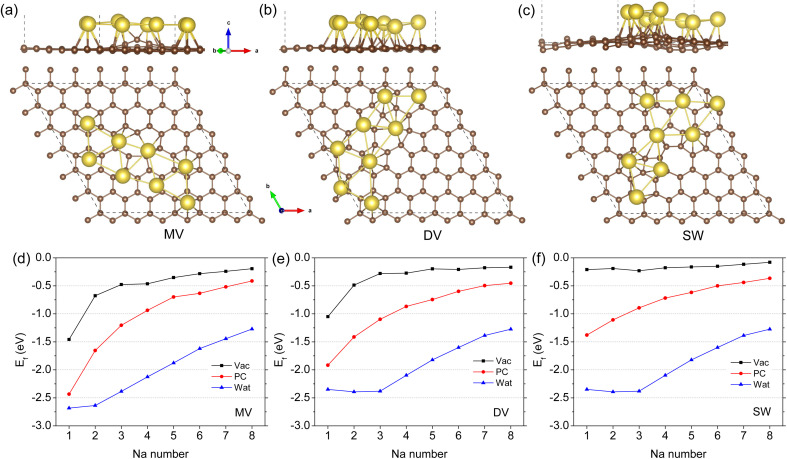
Optimized structures of Na arrangements with 8 Na atoms formed on the defective graphene sheets with a point defect of (a) MV, (b) DV, and (c) SW in side and top views under the vacuum conditions, and (d–f) the corresponding formation energies as a function of Na number under the vacuum, PC and water conditions.

Fig. 3(a)–(c) shows the optimized structures of 8 Na atoms adsorbed on the defective graphene sheet under the vacuum conditions (see Fig. S4–S12† for those of different Na atoms under the vacuum, PC and water conditions). It should be noted that numbers of initial configurations with one Na atom at the N1 hollow site were considered and subsequently optimized to pick out the lowest energy configuration. When the number of Na atoms were smaller than 5, the Na atoms tended to form oligomers like dimer, trimer, and tetramer on the MV and SW defective graphene sheets under the vacuum and PC conditions (see Fig. S4, S5, S10 and S11†) while to be scattered on the DV defective graphene (see Fig. S7 and S8†). For the larger number of Na atoms over 5, the Na atoms were found to be adsorbed in one layer with arranging in two lows on the MV, DV, and SW defective graphene sheets under the vacuum and PC conditions. This is somewhat different from the previous finding that the Na atoms form a cluster with two or three layers on the defective graphene.^38,40^ However, we confirmed that the formation energies of the Na clusters in multiple layers are the almost same to those of the Na arrangements in one layer, indicating no problem of one layer formation. For the cases of aqueous environment, the Na atoms behaved rather disorderly being further away from the graphene sheet compared with the cases of vacuum and PC environments (Fig. S6, S9 and S12†), which can be ascribed to the stronger effect of water.

Fig. 3(d)–(f) presents the formation energies of the Na arrangements as a function of Na atom number for the MV, DV, and SW defective graphene sheets. It turned out that the adsorption of multiple Na atoms up to 8 on the defective graphene is stable under the vacuum, PC, and water conditions owing to their negative formation energies. The stability of multiple Na adsorption on the three kinds of defective graphene weakened gradually as increasing the number of Na atoms. In accordance with the cases of one Na atom adsorption, the formation energies under the PC and water solvent conditions were larger in magnitude, confirming the enhancing effect of electrolyte on sodiation of HC. Through the analysis of electron density differences upon adsorption, it was found that the valence electrons of Na atoms were transferred to the carbon rings, and the charge transfer occurred more severely under the PC electrolyte compared with the vacuum and water conditions (see Fig. S13† for 3 Na atoms).

Then, the electrode potential *versus* Na/Na^+^ counter electrode was estimated to identify the associated charge/discharge region between the slope and plateau regions. If the *x*Na^+^ ions are further adsorbed on the graphene sheet with *n* − *x* adsorbed Na atoms, denoted as (*n* − *x*)Na-G, to form a new structure with formula *n*Na-G, the average electrode potential can be calculated using the following equation,^38^3
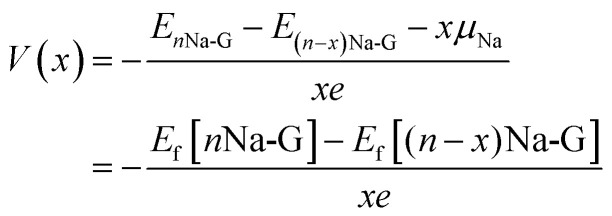
where *E* and *E*_f_ are the total energy and the formation energy of each Na-adsorbed graphene, respectively, and *e* is the elementary charge. We drew the plot of electrode potential as a function of specific capacity, being estimated by *S* = *xeN*_A_/(*M* × *c*) with the Avogadro number *N*_A_, the molecular weight *M* of substrate graphene C_*n*_ per Na atom and the constant *c* = 3.6 mA h C^−1^. When drawing the figure, we fitted the calculation data to the proper functions such as power function, exponential function and cubic polynomial as supported in the data manipulation tools (see Table S1† for fitting functions and parameters).

**Fig. 4:**
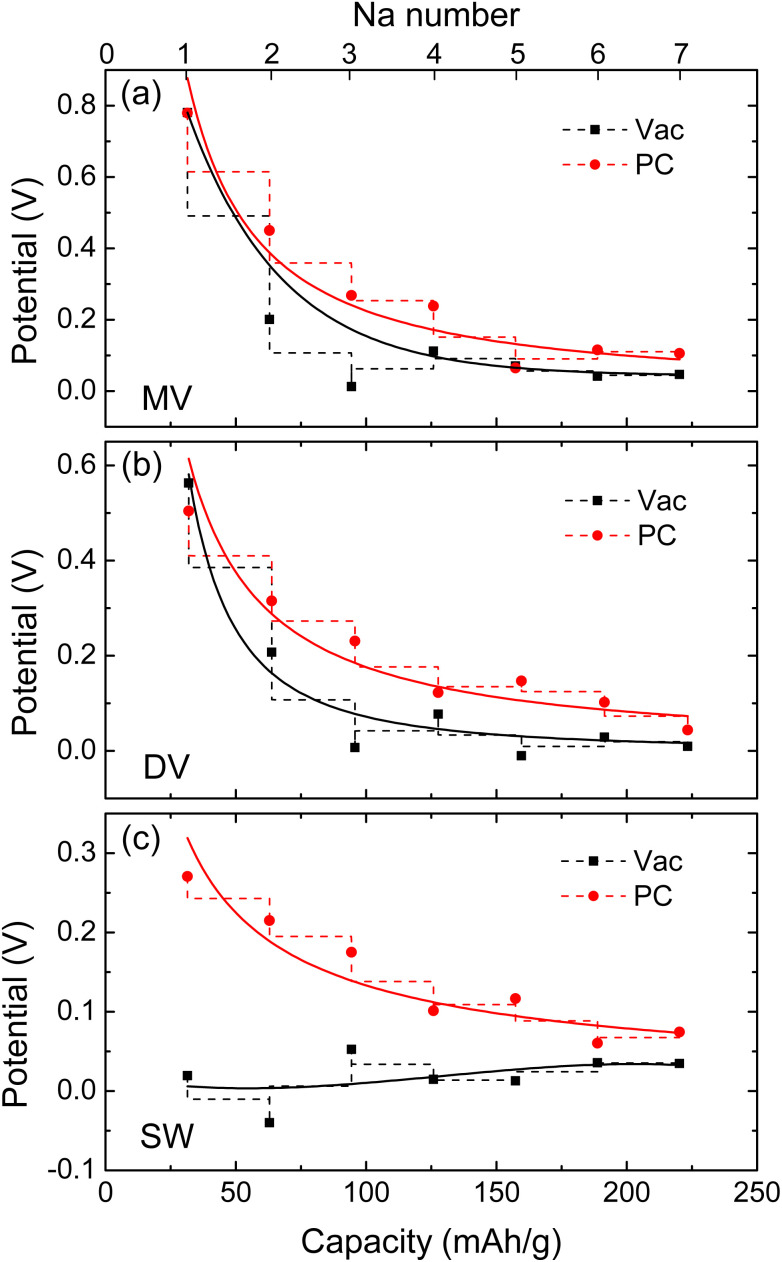
The electrode potential *vs.* Na/Na^+^ as a function of specific capacity with increasing number of adsorbed Na atoms on (a) MV, (b) DV, and (c) SW defective graphene sheets under the vacuum and PC conditions. The solid lines present the fitting lines to the power functions.

Fig. 4 shows the electrode potential curves estimated as a function of specific capacity under the vacuum and PC conditions. For the cases of water conditions, the calculated electrode potentials were found to be not reasonable, which can be expected from the improper geometries of Na adsorption. Under the PC solvent environment, the MV, DV and SW defective graphene sheets exhibited the voltage profiles (red-colored curves) with the identical lowest voltage values of 0.1 V at the specific capacity of about 200 mA h g^−1^ and the different highest voltage values of 0.8, 0.6, and 0.3 V at the specific capacity of about 30 mA h g^−1^. These curves are well associated with the slope region when compared with the experimental discharge curves. Under the vacuum conditions, the MV and DV defective graphene sheets also showed the voltage profiles well suited to the slope region with the lowest voltage below 0.1 V, but the SW case did not show the reasonable discharge curve. Therefore, it turned out that the adsorption of multiple Na atoms on the MV and DV defective graphene sheets are responsible for the slope region of the charge/discharge curves in HC. We especially emphasize that the consideration of electrolyte effect like PC is important for reproducing the slope part of the experimental discharge curve. Our finding is agreed well with the previous viewpoint that the Na adsorption on defective pore wall is related with the slope region.^38^ Although not considered in this work, we expect that the intercalation of Na atoms into the space between the graphene sheets is related with the plateau part around 0.1 V over 200 mA h g^−1^ capacity.

**Fig. 5:**
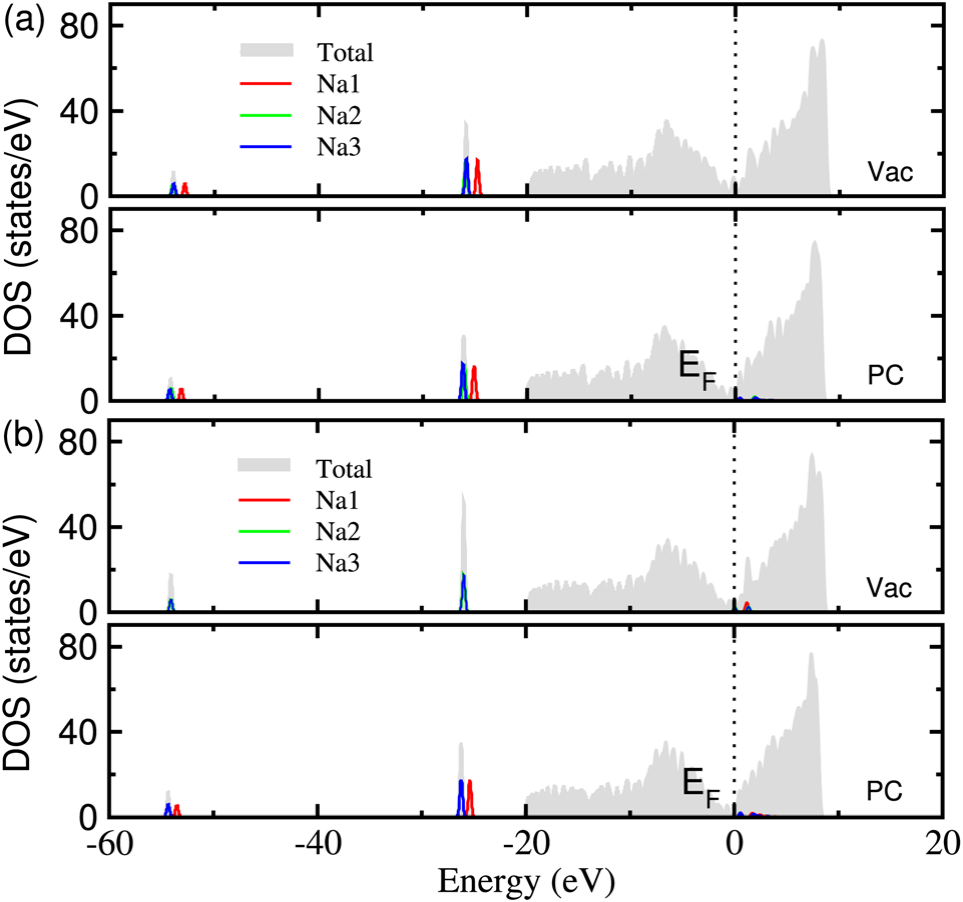
The electronic density of states (DOS) in (a) MV and (b) DV defective graphene sheets with 3 adsorbed Na atoms, calculated under the vacuum and PC conditions.

In order to clarify the electronic conductance, which is one of the key properties of electrode material, we calculated the electronic density of states (DOS) of Na adsorbed graphene. Fig. 5 shows the calculated DOS of the MV and DV defective graphene with 3 adsorbed Na atoms under the vacuum and PC conditions. For all the cases, one can see no band gap in the total DOS, which is mainly attributed to the graphene sheet, indicating a good electronic conductance in the planar direction. A small amount of electronic states are seen in the conduction band region near the Fermi level *E*_F_ for the MV defect under the PC solvent and the DV defect under vacuum and PC conditions. This indicates that the Na atoms release the valence electron to become Na^+^ ions in accordance with the analysis of the electronic density difference.

## 4 Conclusions

In this work, we have investigated the adsorption of Na atoms on the defective graphene sheets by using the first-principles calculations within the framework of joint density functional theory (JDFT), aiming at clarifying the sodiation mechanism of hard carbon (HC) in contact with the liquid organic electrolyte. We made 6 × 6 × 1 supercell models with a point defect such as mono-vacancy (MV), di-vacancy (DV), and Stone–Wales (SW) for defective graphene sheet, and simulated the adsorption of one Na atom and subsequently multiple Na atoms up to 8 on the sheet under the propylene carbonate (PC) and water solvent as well as vacuum conditions. Within the JDFT framework, the nonlinear polarizable continuum model (PCM) and the charge-asymmetric nonlocally-determined local electric (CANDLE) solvation model were adopted for PC and water solvents, respectively. Our calculations revealed that the most favourable site for one Na atom adsorption on the defective graphene is the central site of each point defect due to the lowest adsorption energy among the possible different sites. The effect of PC and water solvent was verified by observing the lowering of adsorption energy and the water solvent with larger bulk dielectric constant was found to have further effect on Na adsorption than PC. The electronic density difference upon the Na adsorption on the graphene sheet was considered, finding a valence electron transfer from Na to graphene and its enhancement by solvent effect. Furthermore, we demonstrated the stability of multiple Na adsorption up to 8 atoms on the defective graphene with a formation of oligomers and arrangements, confirming the positive effect of PC and water solvent on the adsorption. Using the formation energies of Na aggregates, the electrode potential of each defective graphene *versus* Na/Na^+^ counter electrode was estimated, verifying that the multiple Na adsorption of the MV and DV defective graphene under PC electrolyte conditions is related with the slope region of discharge curve in HC. We believe this work contributes to clarifying the sodiation mechanism of HC-based anode and thus optimizing the anode and electrolyte for sodium-ion batteries.

## Author contributions

Chol Ryu performed the calculation and post-processing of calculation results. Song-Bom Rim and Yong Kang assisted with the DFT calculations, and contributed to useful discussions. Chol-Jun Yu developed the original project, drafted the first manuscript, and supervised the work. All authors reviewed the manuscript.

## Conflicts of interest

There are no conflicts to declare.

## Supplementary Material

RA-013-D2RA08168G-s001
