# Supplementary material for: First-principles study of sodium adsorption on defective graphene under propylene carbonate electrolyte conditions
Source: RSC Adv. 2023 Feb 14;13(9):5627–33. doi: 10.1039/d2ra08168g (PMC9926951; doi:10.1039/d2ra08168g)
Supplement: RA-013-D2RA08168G-s001 [file RA-013-D2RA08168G-s001.pdf]

Electronic Supplementary Material (ESI) for RSC Advances.
This journal is © The Royal Society of Chemistry 2023

Supporting Information – First-principles study of sodium adsorption on defective graphene under propylene carbonate electrolyte conditions

Chol Ryu, Song-Bom Rim, Yong Kang and Chol-Jun Yu*

Chair of Computational Materials Design, Faculty of Materials Science, Kim Il Sung University, Ryongnam-Dong, Taesong District, Pyongyang, PO Box 76, Democratic People's Republic of Korea

Table S1. Fitting functions and determined fitting parameters for electrode potentials.

Defects	Conditions	Fitting functions	Fitting parameters	Adjusted R^2
MV	Vacuum	$y = a + b\exp(-(x-c)/d)$	$a = 0.04242$, $b = 0.73808$, $c = 31.46143$, $d = 36.31263$	0.7779
	PC	$y = ax^b$	$a = 50.8751$, $b = -1.1773$	0.9258
DV	Vacuum	$y = ax^b$	$a = 329.5624$, $b = -1.8310$	0.9531
	PC	$y = ax^b$	$a = 27.1105$, $b = -1.0938$	0.8558
SW	Vacuum	$y = a + bx + cx^2 + dx^3$	$a = 0.0191$, $b = -6.2445 \cdot 10^{-4}$, $c = 7.2459 \cdot 10^{-5}$, $d = -1.8745 \cdot 10^{-8}$	0.6290
	PC	$y = ax^b$	$a = 4.3205$, $b = -0.7554$	0.8220

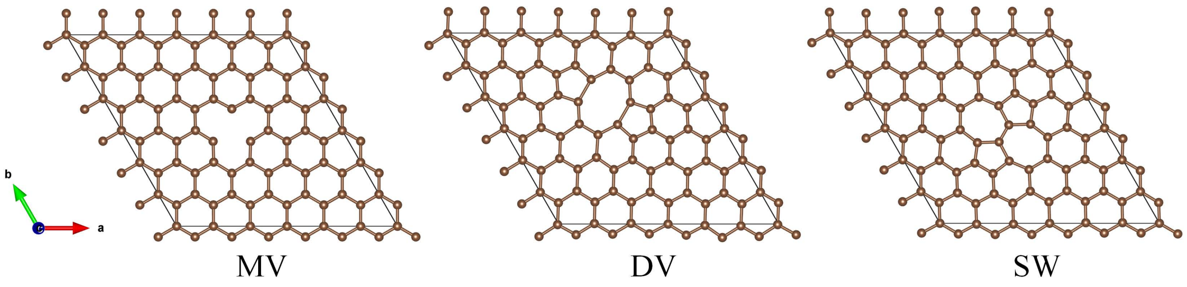

Figure S1. Optimized structure of supercells for defective graphene sheets including a point defect such as mono-vacancy (MV), di-vacancy (DV) and Stone-Wales (SW).

*cj.yu@ryongnamsan.edu.kp

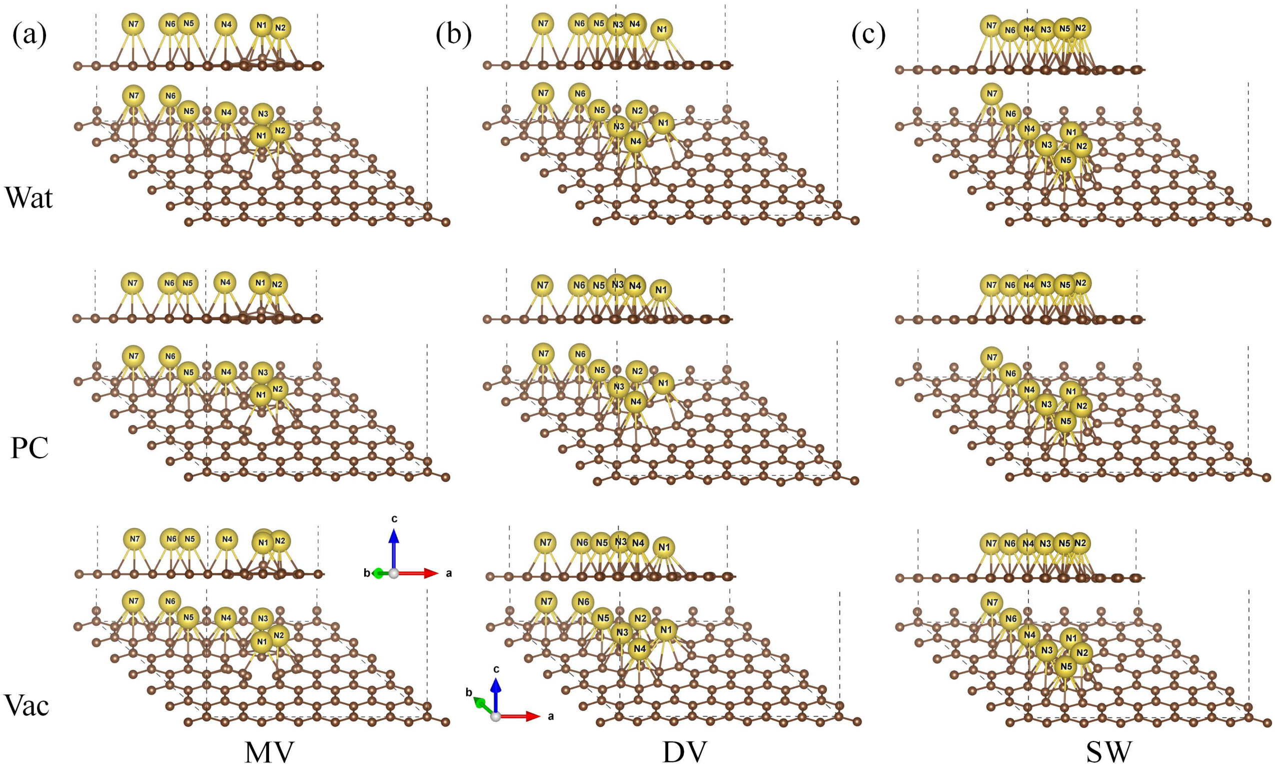

Figure S2. Ball-and-stick view for Na-adsorbed defective graphene sheets optimized under vacuum, PC and water condition. Different Na sites are denoted as N1–N7.

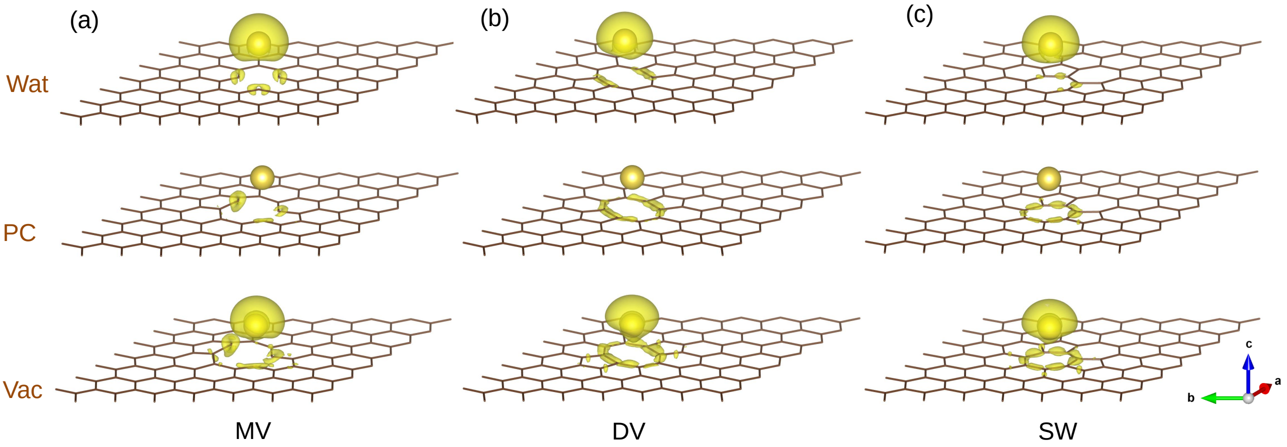

Figure S3. Isosurface view of the electronic density differences at the value of 0.0015 $|e|/Å^3$ upon Na adsorption at the N1 hollow sites over the defective graphene with (a) MV, (b) DV, and (c) SW defects in the vacuum, PC and water solvent environments. Only the electron depletion is shown.

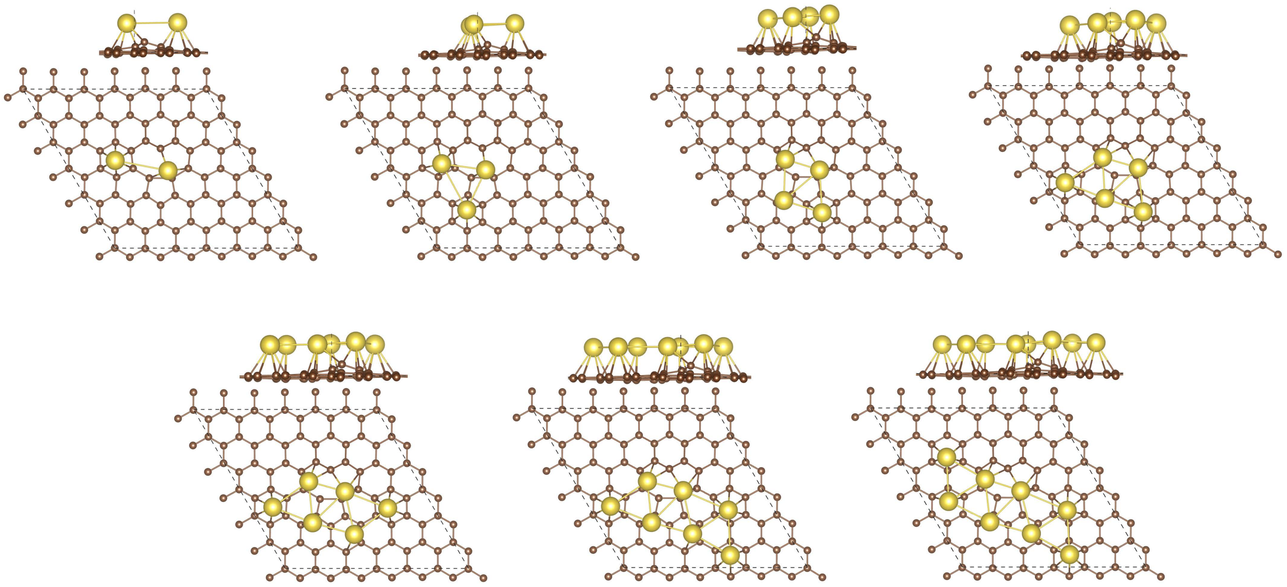

Figure S4. Ball-and-stick view for the optimized structures of multiple Na atoms (2–8) on the MV defective graphene under the vacuum condition.

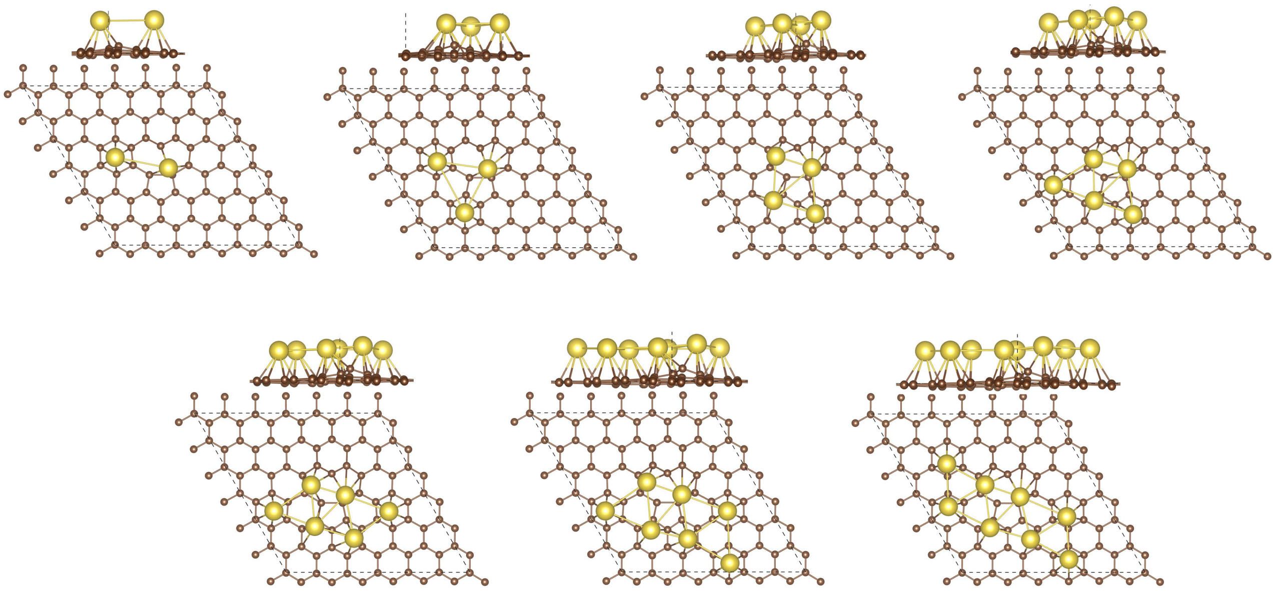

Figure S5. Ball-and-stick view for the optimized structures of multiple Na atoms (2–8) on the MV defective graphene under the PC condition.

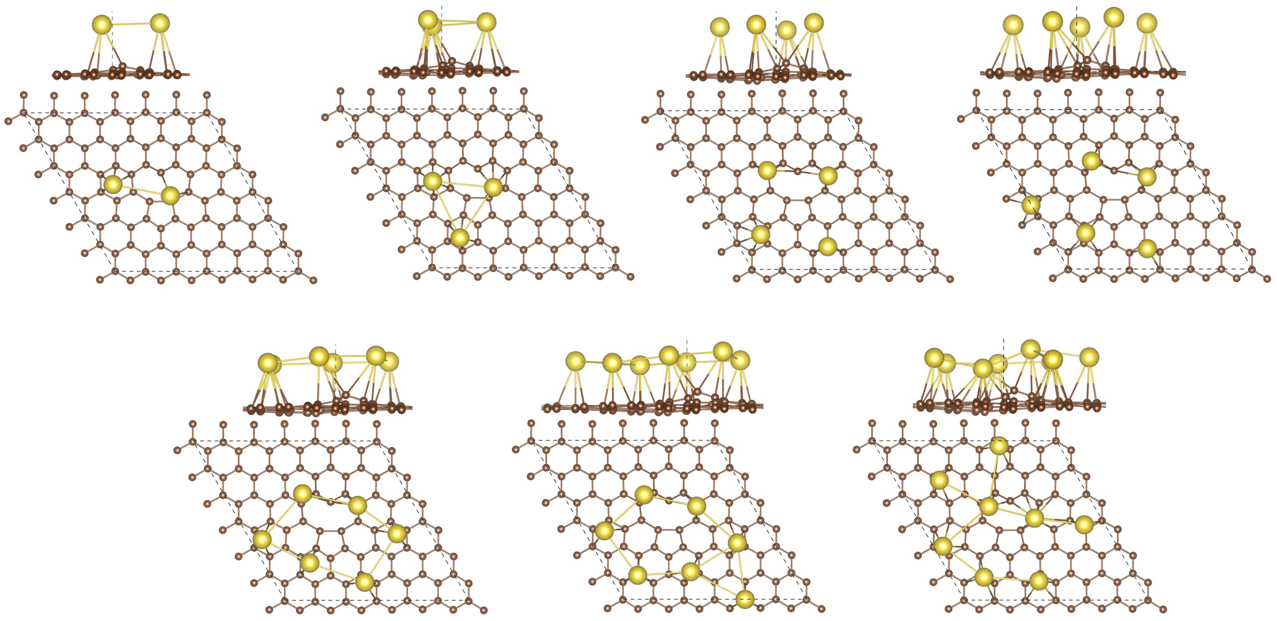

Figure S6. Ball-and-stick view for the optimized structures of multiple Na atoms (2–8) on the MV defective graphene under the water condition.

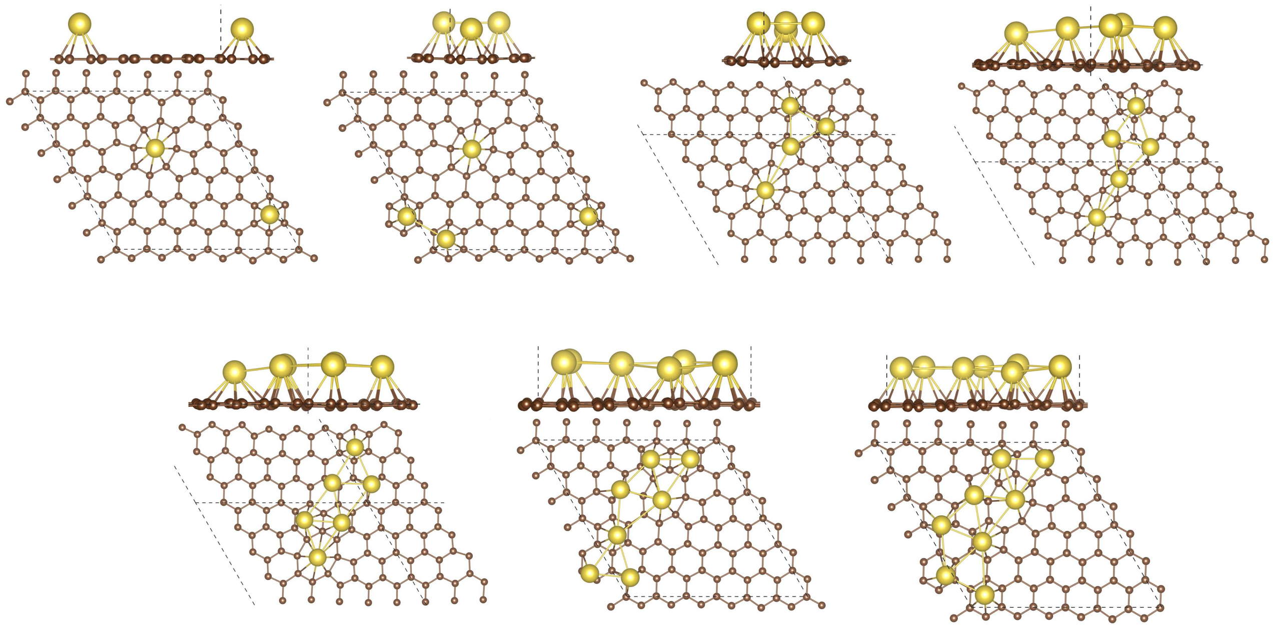

Figure S7. Ball-and-stick view for the optimized structures of multiple Na atoms (2–8) on the DV defective graphene under the vacuum condition.

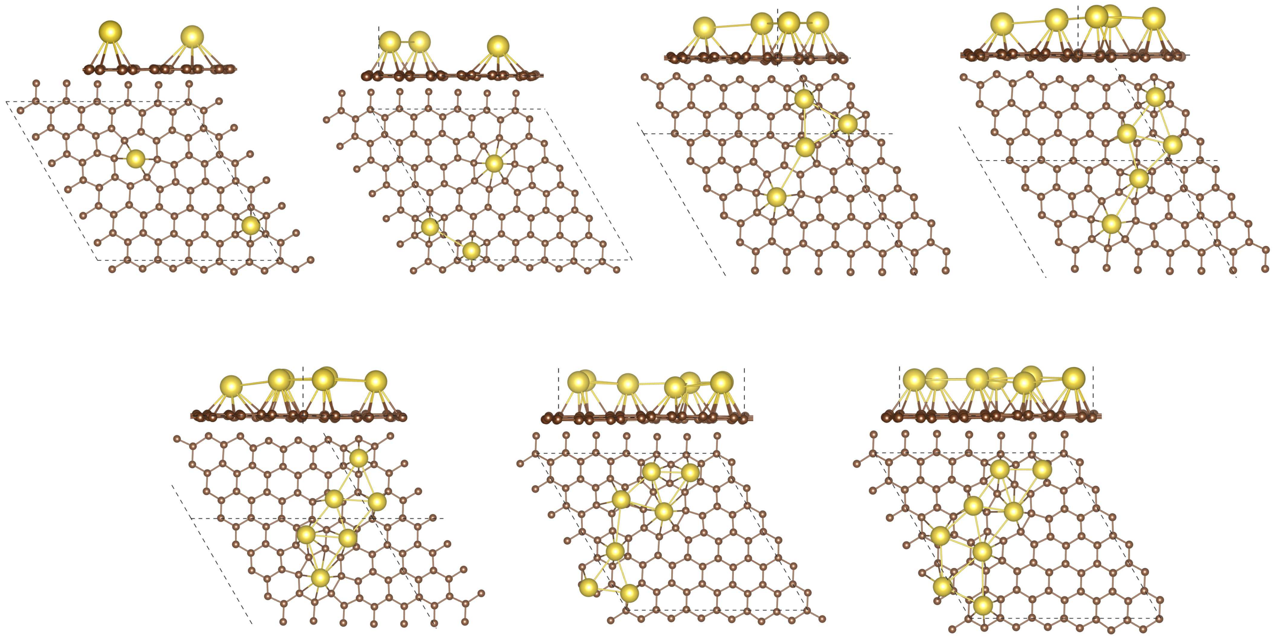

Figure S8. Ball-and-stick view for the optimized structures of multiple Na atoms (2–8) on the DV defective graphene under the PC condition.

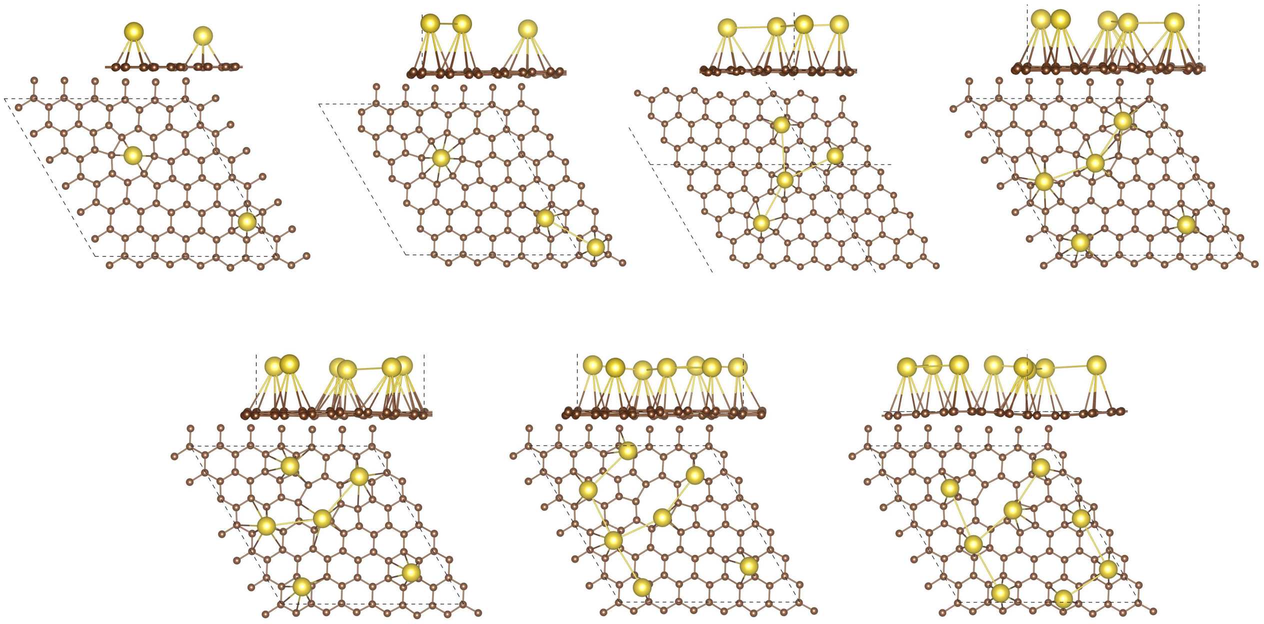

Figure S9. Ball-and-stick view for the optimized structures of multiple Na atoms (2–8) on the DV defective graphene under the water condition.

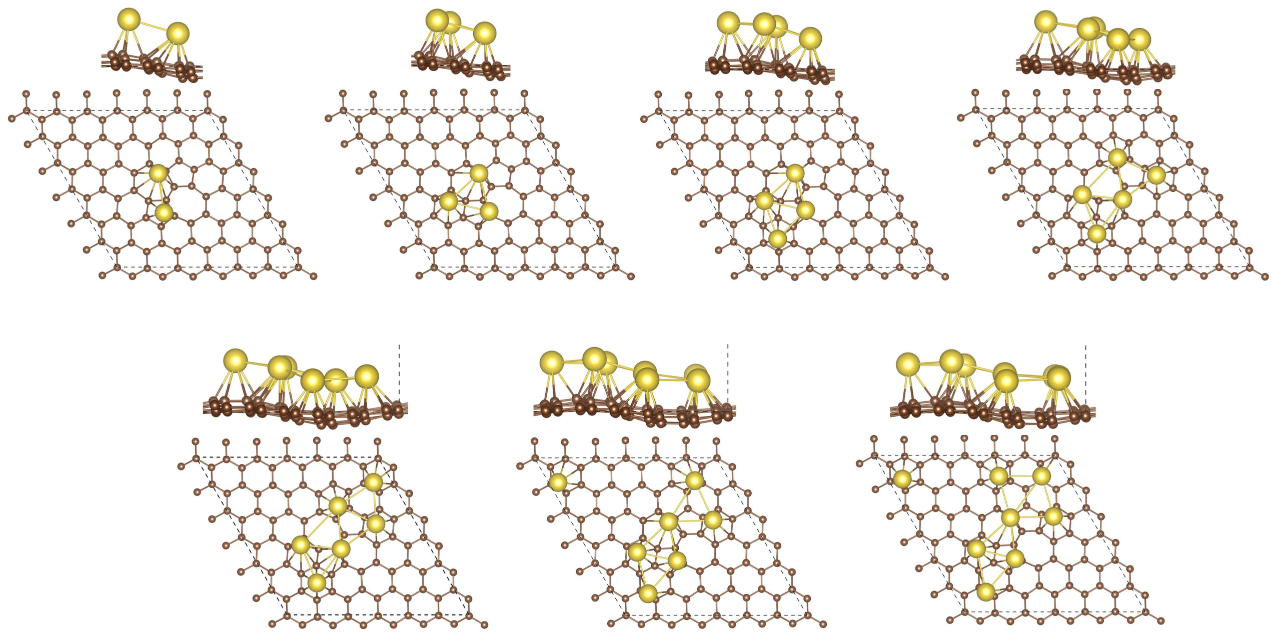

Figure S10. Ball-and-stick view for the optimized structures of multiple Na atoms (2–8) on the SW defective graphene under the vacuum condition.

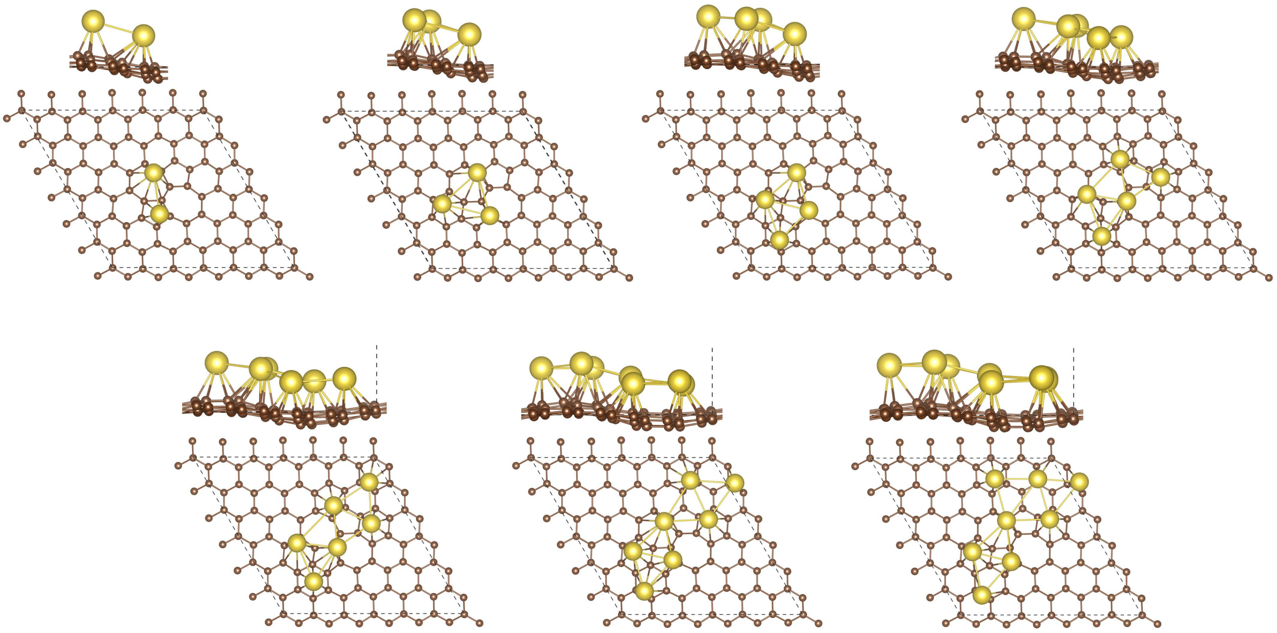

Figure S11. Ball-and-stick view for the optimized structures of multiple Na atoms (2–8) on the SW defective graphene under the PC condition.

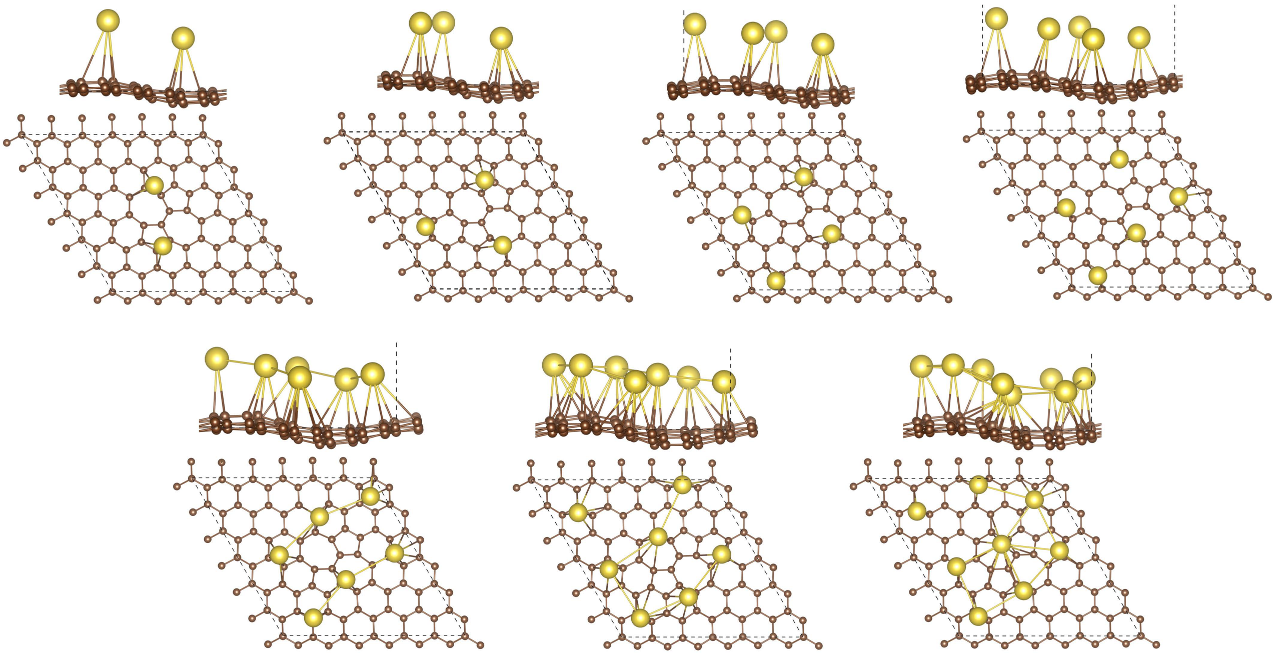

Figure S12. Ball-and-stick view for the optimized structures of multiple Na atoms (2–8) on the SW defective graphene under the water condition.

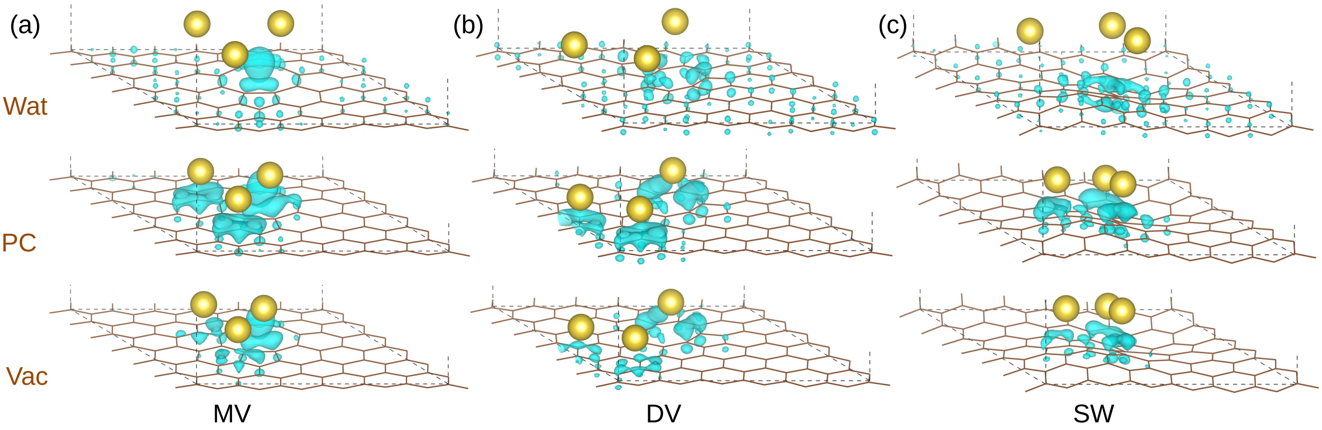

Figure S13. Isosurface view of the electronic density differences at the value of 0.0015 $|e|/\text{Å}^3$ upon 3 Na adsorption on the defective graphene with (a) MV, (b) DV, and (c) SW defect in the vacuum, PC and water solvent environment. Only the electron accumulation is shown.

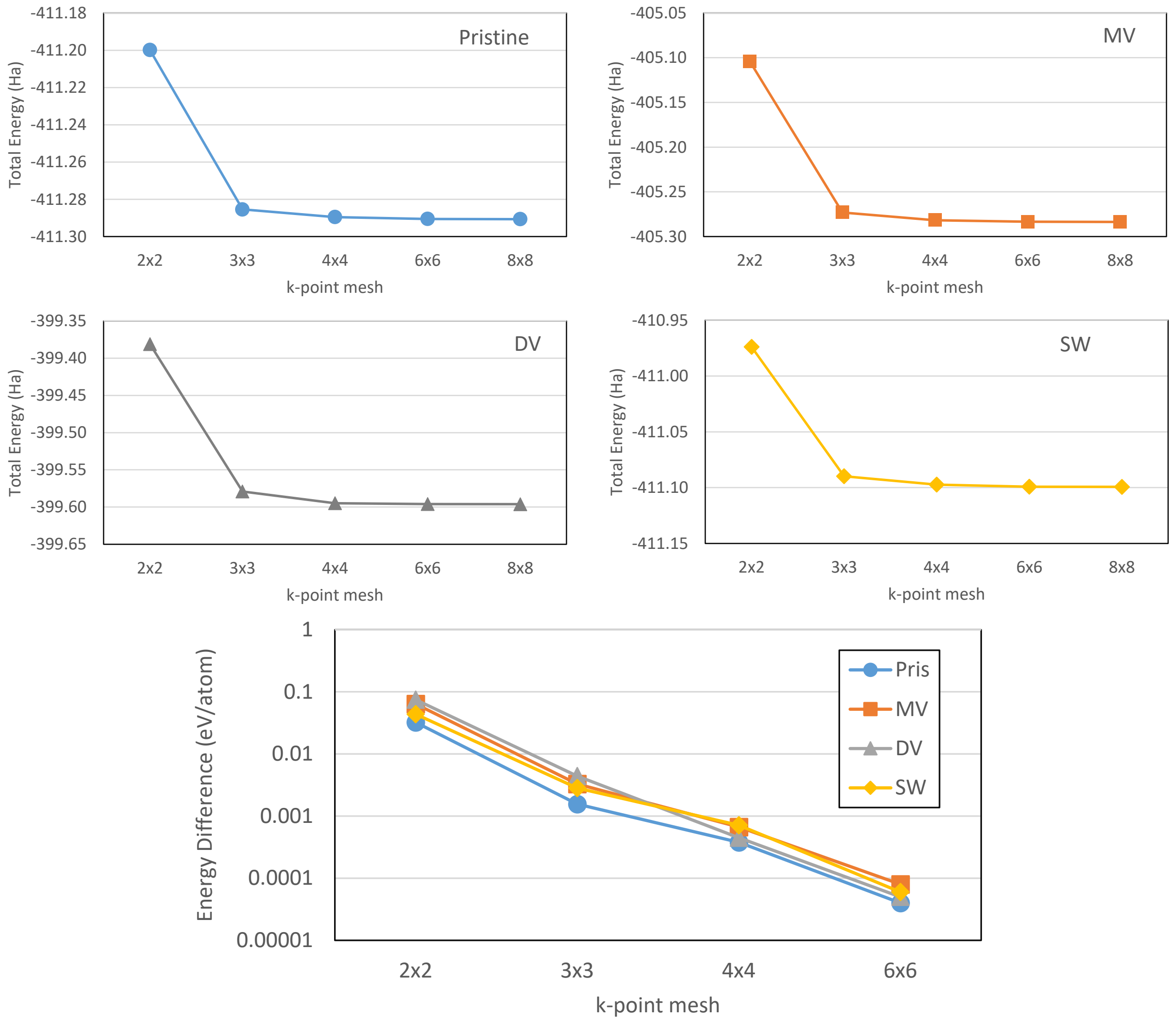

Figure S14. Total energy convergence test with respect to the k-point mesh.
